# Novel Muscle Imaging in Inflammatory Rheumatic Diseases—A Focus on Ultrasound Shear Wave Elastography and Quantitative MRI

**DOI:** 10.3389/fmed.2020.00434

**Published:** 2020-08-12

**Authors:** Matthew Farrow, John Biglands, Abdulrahman M. Alfuraih, Richard J. Wakefield, Ai Lyn Tan

**Affiliations:** ^1^Leeds Institute of Rheumatic and Musculoskeletal Medicine, Chapel Allerton Hospital, University of Leeds, Leeds, United Kingdom; ^2^NIHR Leeds Biomedical Research Centre, Leeds Teaching Hospitals NHS Trust, Leeds, United Kingdom; ^3^School of Pharmacy and Medical Sciences, University of Bradford, Bradford, United Kingdom; ^4^Medical Physics and Engineering, Leeds Teaching Hospitals NHS Trust, Leeds, United Kingdom; ^5^Radiology and Medical Imaging Department, Prince Sattam Bin Abdulaziz University, Al-Kharj, Saudi Arabia

**Keywords:** muscle, myositis, myopathy, MRI, ultrasound, shear wave elastography, imaging, rheumatic

## Abstract

In recent years, imaging has played an increasing role in the clinical management of patients with rheumatic diseases with respect to aiding diagnosis, guiding therapy and monitoring disease progression. These roles have been underpinned by research which has enhanced our understanding of disease pathogenesis and pathophysiology of rheumatology conditions, in addition to their key role in outcome measurement in clinical trials. However, compared to joints, imaging research of muscles is less established, despite the fact that muscle symptoms are very common and debilitating in many rheumatic diseases. Recently, it has been shown that even though patients with rheumatoid arthritis may achieve clinical remission, defined by asymptomatic joints, many remain affected by lingering constitutional systemic symptoms like fatigue, tiredness, weakness and myalgia, which may be attributed to changes in the muscles. Recent improvements in imaging technology, coupled with an increasing clinical interest, has started to ignite new interest in the area. This perspective discusses the rationale for using imaging, particularly ultrasound and MRI, for investigating muscle pathology involved in common inflammatory rheumatic diseases. The muscles associated with rheumatic diseases can be affected in many ways, including myositis—an inflammatory muscle condition, and myopathy secondary to medications, such as glucocorticoids. In addition to non-invasive visual assessment of muscles in these conditions, novel imaging techniques like shear wave elastography and quantitative MRI can provide further useful information regarding the physiological and biomechanical status of the muscle.

## Introduction

Advances in diagnostic imaging in rheumatology, particularly in the area of arthritis, have contributed to significant clinical benefits to patients and improved knowledge in disease pathogenesis. Despite the usefulness of ultrasound and magnetic resonance imaging (MRI) in diagnosing arthritis and monitoring disease progression in joints and related joint structures, the role of muscle imaging has conventionally been centered around the diagnosis of inflammatory muscle diseases. However, with an increasing appreciation of the impact and prevalence of muscular symptoms in rheumatic diseases (1), and as a result of technological developments, recent attention has been directed toward the utility of imaging for the assessment of muscle pathology in rheumatic diseases.

The impact of muscle weakness is significant for the health of patients and is associated with disease activity (2). There is an unmet need for further understanding of more generalized muscle pathology observed in rheumatic diseases. This is required to develop effective future strategies to target this under-researched area. In addition to ultrasound and MRI, positron emission tomography combined with computed tomography (PET-CT) is increasingly used in clinical practice to aid the diagnosis of myositis, with the added advantage that this technique can screen for malignancy and evaluate related pulmonary pathologies (3, 4). This perspective will discuss recent novel imaging developments in ultrasound and MRI for the assessment of muscles in common inflammatory rheumatic diseases, with a particular focus on research applicability of shear wave elastography and quantitative MRI in improving the knowledge of muscle pathology in rheumatic diseases. The potential application of these novel techniques will be explored in the context of three common inflammatory rheumatology conditions where the muscle is of interest. The first is in myositis, a primary inflammatory condition of the muscle; the second is in glucocorticoid-induced myopathy, where patients with giant cell arteritis and polymyalgia rheumatica are at risk from the complications of prolonged high dose steroid therapy; and the third is rheumatoid arthritis where patients often complain of muscle related symptoms in addition to their joints.

## Muscle Imaging Techniques

### Ultrasound

Due to recent innovations, ultrasonography has evolved from demonstrating mainly anatomical details to elucidating the physical properties of tissues. Although B-mode ultrasonography has been shown to be reliable in assessing muscle mass and quality (5–7), and muscle fibers during dynamic scanning (8, 9), recent interest has been directed to a new type of ultrasound called elastography (10). This technique provides a measure of the stiffness of tissue (11). The first generation machines, developed in the 1990's utilized “strain elastography,” where a mechanical ultrasound pulse was generated by repeated probe compressions on the skin by the operator. The returned waves could be used to qualitatively estimate stiffness by comparing the pre- and post-compression tissue deformations. The images were represented as a color map, superimposed on a B-mode image (blue—hard, and red—soft). Shear wave elastography (SWE) has more recently been introduced to offer quantitative measurements by monitoring the velocity of the shear waves generated by strong acoustic pulses. The physics behind shear waves is complex and beyond the scope of this article, but essentially, the velocity of the shear wave increases proportionally with Young's elasticity modulus. SWE is less operator dependent than strain elastography, and offers more objective outcomes. Hence, this perspective will focus on the potential uses of SWE, which has more commonly been established for examining breast, liver, thyroid and prostate tissues (12–14).

In the musculoskeletal setting, SWE has largely been used to study tendinopathies (15, 16). More recently however, SWE has been extended to examining muscles, and has been shown to be a reliable tool to measure muscle stiffness (17–19). The technique has been used in the sports and exercise scenarios, to assess muscle injuries and the effect of exercise interventions on muscles (20, 21). Clinically, SWE of muscles, such as of the rotator cuff muscles that are commonly susceptible to tears, has been shown to inform appropriate management strategies (22). In the hospital setting, it has shown good reliability for monitoring the muscles of critically ill patients (23). Other clinical uses of SWE are in the assessment of the muscles in neuromuscular conditions including Parkinson's disease, Duchene muscular dystrophy, and in post-stroke spasticity (24–26). Insight into the potential of using SWE in assessing muscle elasticity has prompted recommendations into standardizing the technique for optimal data acquisition (27–29). It is known that muscles change with age, which is apparent in the structure and the function of the muscles (30, 31). Although some studies using SWE have shown that there is a decline in muscle stiffness with age (32–35), this observation was not corroborated by others (36–38). These studies looked at different muscles, which may have influenced the final outcomes, as it has been found that SWE findings may be muscle-dependent (39).

### MRI

MRI offers the ability to examine deeper tissue structures compared to ultrasound. Although MRI can also measure the elasticity of muscles using magnetic resonance elastography (40–44), the cost of the technique is more prohibitive when compared to SWE; thus far, the utility of MRI in assessing muscle elasticity is still debatable (45).

Due to its excellent spatial and contrast resolution, MRI can evaluate a wide array of muscle pathologies including muscle injury (46) and soft tissue masses (47). MRI is beginning to have a role in the diagnosis and monitoring of muscle disease and in guiding muscle biopsy (48, 49). Whole-body MRI can help identify muscular involvement over large anatomical regions (50, 51). Aside from conventional MRI there is also an important role for quantitative MRI (qMRI) measurements, such as fat fraction, T2 measurement and diffusion tensor imaging (DTI), in muscle imaging. Quantitative MRI can provide information about tissue microstructure that may not be apparent in conventional MRI. It provides objective measurements, as opposed to a qualitative assessment and has been shown to be reliable and reproducible in the muscle (52, 53).

Fat fraction measurements exploit the differences in the resonant frequencies between the MR signals of fat and water in order to generate a measurement of the proportion of fat in each voxel in the image (54). These measurements provide an objective assessment of fatty infiltration in muscle, which is a common pathology in muscle disease.

Measurements of the T2-relxation time also have applications in the muscle. T2, or the spin-spin relaxation time, is one of the fundamental contrast mechanisms in MRI. By measuring the signal at multiple echo times, measurements of T2 can be made within the muscle. Raised T2 is often interpreted as increased fluid due to edema or inflammation. However, care must be taken in the interpretation of T2. Fat can also increase T2 values and fat suppression is challenging in T2 measurements (55, 56), with some papers arguing that T2 may actually decrease with disease activity (57).

Diffusion MRI is able to measure water diffusion in the muscle. Diffusion measurements in inflamed muscle may be greater due to increased fluid in the extracellular space. Diffusion tensor imaging (DTI) allows the anisotropy of the diffusion to be assessed. As muscle is made up of long fibers, or fibrils, muscle diffusion is highly anisotropic and ordered. As muscle diameters are relatively wide, long diffusion times are necessary if the measurements are to be sensitive to restricted diffusion across the fiber. Fiber disorganization and deterioration through trauma or disease can be detected by DTI measurements, such as fractional anisotropy (FA) (26). However, the interpretation of what a change in diffusion measurement means is difficult. Fiber disorder, fiber density, fiber diameter (58, 59) and changes in extracellular water (60) can all affect diffusion parameters. There is on-going research into the use of modeling to analyse DTI acquisitions at multiple diffusion times to separate out different properties of the muscle microstructure from diffusion measurements (61–63).

In the clinical setting, qMRI of various tissues including muscles shows potential as a promising biomarker for assessing and monitoring a range of neuromuscular and musculoskeletal diseases (57, 64–68). In general, these patients show higher muscle fat fractions, smaller muscle volume, and increased T2 measures, which also correlate with muscle function (69).

## Muscle Imaging in Inflammatory Rheumatic Diseases

### Myositis

The idiopathic inflammatory myopathies (IIM) are the commonest inflammatory muscle diseases seen by rheumatologists. They are a heterogeneous group of autoimmune inflammatory muscle conditions comprising mainly of dermatomyositis and polymyositis, which present with muscle weakness, raised muscle enzymes, abnormal electromyography (EMG), abnormal muscle biopsies and myositis-related antibodies (70).

**Figure 1:**
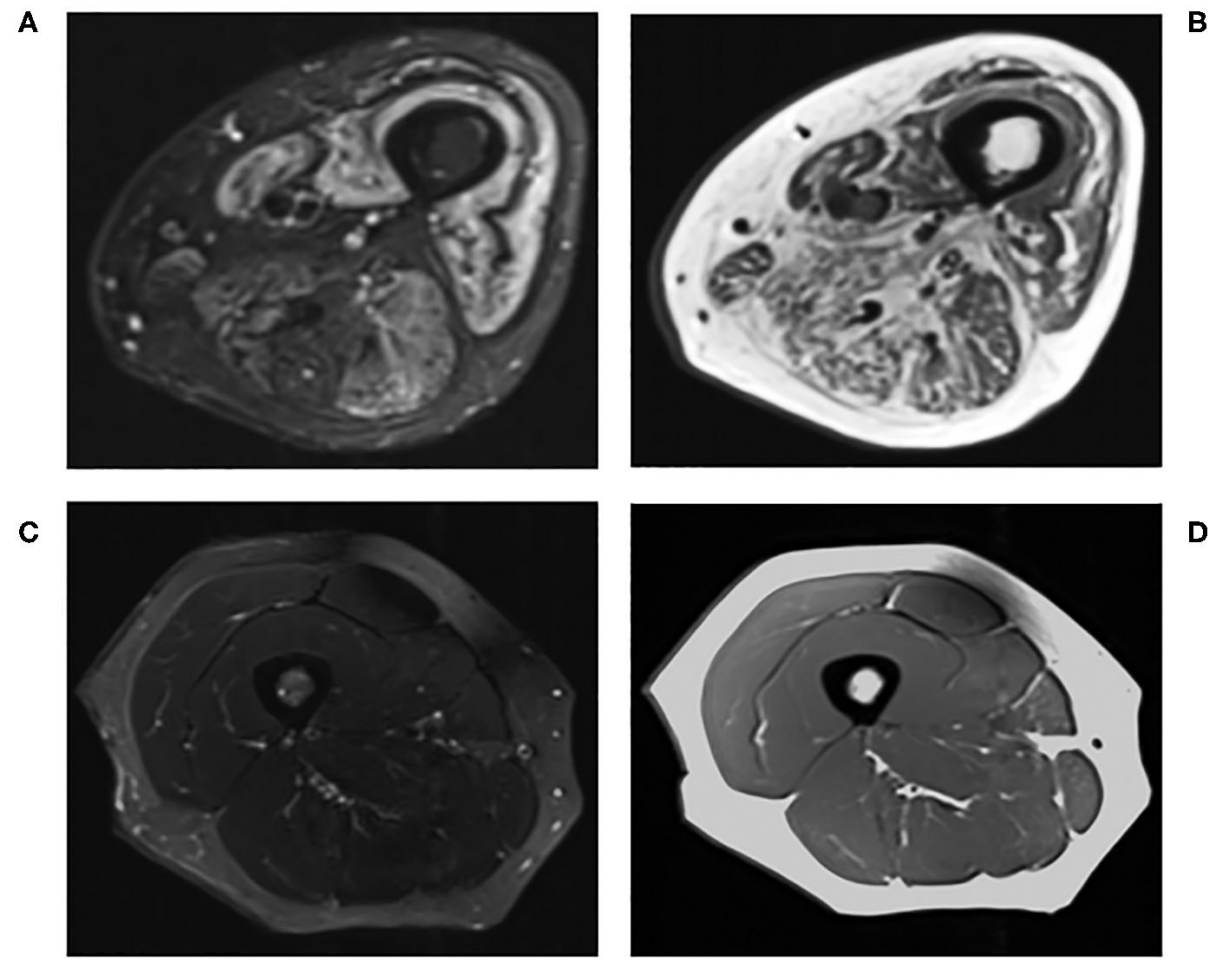
Conventional MRI of the right thigh in **(A)** T2-STIR and **(B)** T1-weighted images of a 60-years-old male with active myositis, compared to **(C)** T2-STIR and **(D)** T1-weighted images of a 45-years-old healthy female.

MRI has become an integral imaging tool in the clinical diagnosis and monitoring of disease activity of myositis due to its ability to non-invasively detect abnormal muscles and identify the most suitable site for muscle biopsies (Figure 1) (45, 71–74). Reassuringly, MRI findings in myositis correlate well with biopsy results (75), and whole body MRI can be more sensitive than muscle enzymes and EMG in diagnosing myositis (76). Nevertheless, the image interpretation can be subjective (77), there is no validated MRI protocol for assessing myositis (45) and MRI findings in isolation may not be specific enough for diagnostic purposes (78).

**Figure 2:**
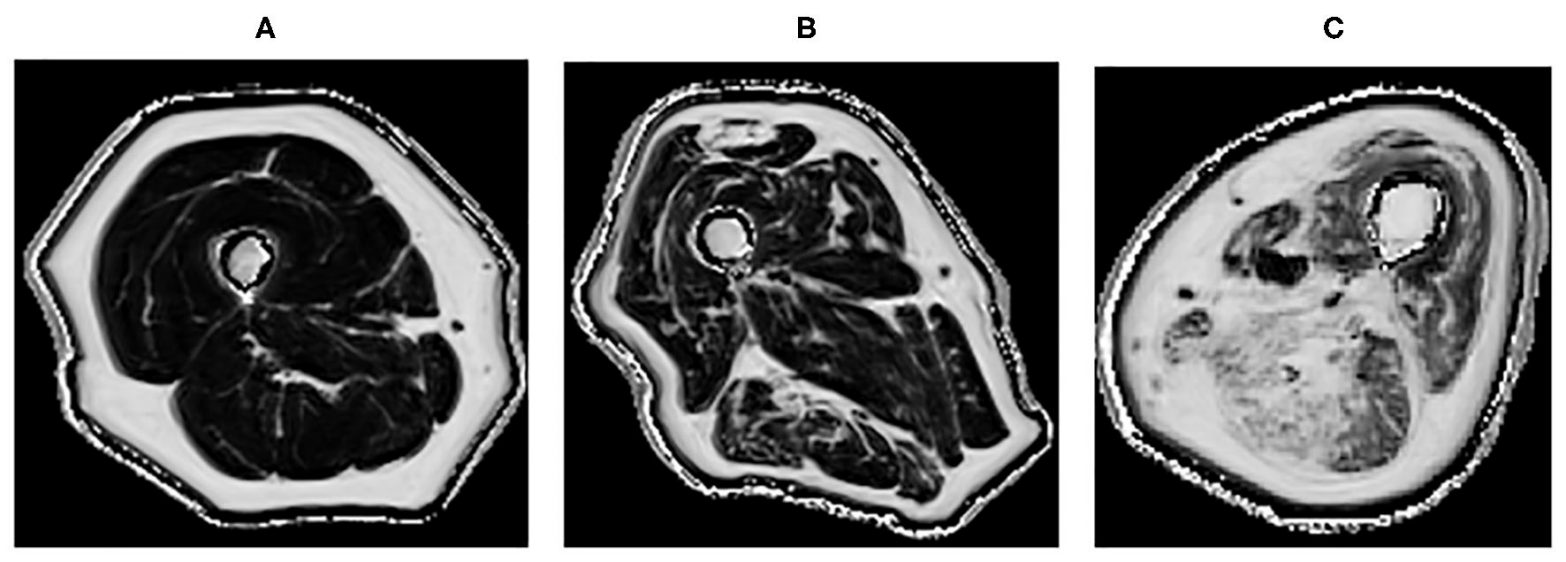
Quantitative MRI fat fraction measurement in the quadriceps and hamstrings, respectively in the thigh in **(A)** 45-years-old healthy female with a fat fraction of 1.9 and 2.7%, respectively, **(B)** 83-years-old healthy male presenting with fatty infiltration associated with healthy aging with a fat fraction of 9.6 and 13.4%, respectively, **(C)** 60-years-old male with active myositis presenting with fatty infiltration with a fat fraction of 19.6 and 28.5%, respectively.

Quantitative MRI, which allows further characterization of the muscle structure at a microscopic level, can provide a more precise description of muscle pathology (55, 73, 79). It could potentially be used in longitudinal monitoring of disease (80). It has been demonstrated that T2 and fat fraction increase in myositis patients (Figure 2), demonstrating that MRI is sensitive enough to quantitatively detect muscle edema (55, 81) and myosteatosis (82). These measures could be used to more accurately guide muscle biopsies. This may be of greater importance in patients with low grade inflammation, where there are subtle muscle changes that might go undetected by conventional MRI. DTI measurements are sensitive to subtle changes in the muscle, and have been used to detect differences in muscle due to diseases including myositis (83). However, muscle DTI is far from standardized. The optimal methods and parameters for performing diffusion in muscle have not been established and larger studies are necessary to establish whether diffusion will be a useful tool for monitoring muscle disease in clinical practice.

One of the drawbacks of MRI as an imaging tool is its cost. Often, this is the deciding factor in choosing ultrasonography over MRI as a more feasible modality in assessing articular joints. But does this cost consideration translate to examining muscles or in patients with myositis? In addition to the more favorable cost compared to MRI, ultrasound also has a greater acceptability by patients. Although there is greater operator dependence for ultrasound, there is the possibility to apply the ultrasound information directly in the clinical setting. There is a suggestion that ultrasound elastography of muscles may be able to aid diagnosis of myositis and its follow-up (84), but the impression is that ultrasound is unlikely to replace MRI in the clinical setting in myositis just yet, because the current evidence is not strong, due to small sample sized studies that results in inconclusive findings (85).

**Figure 3:**
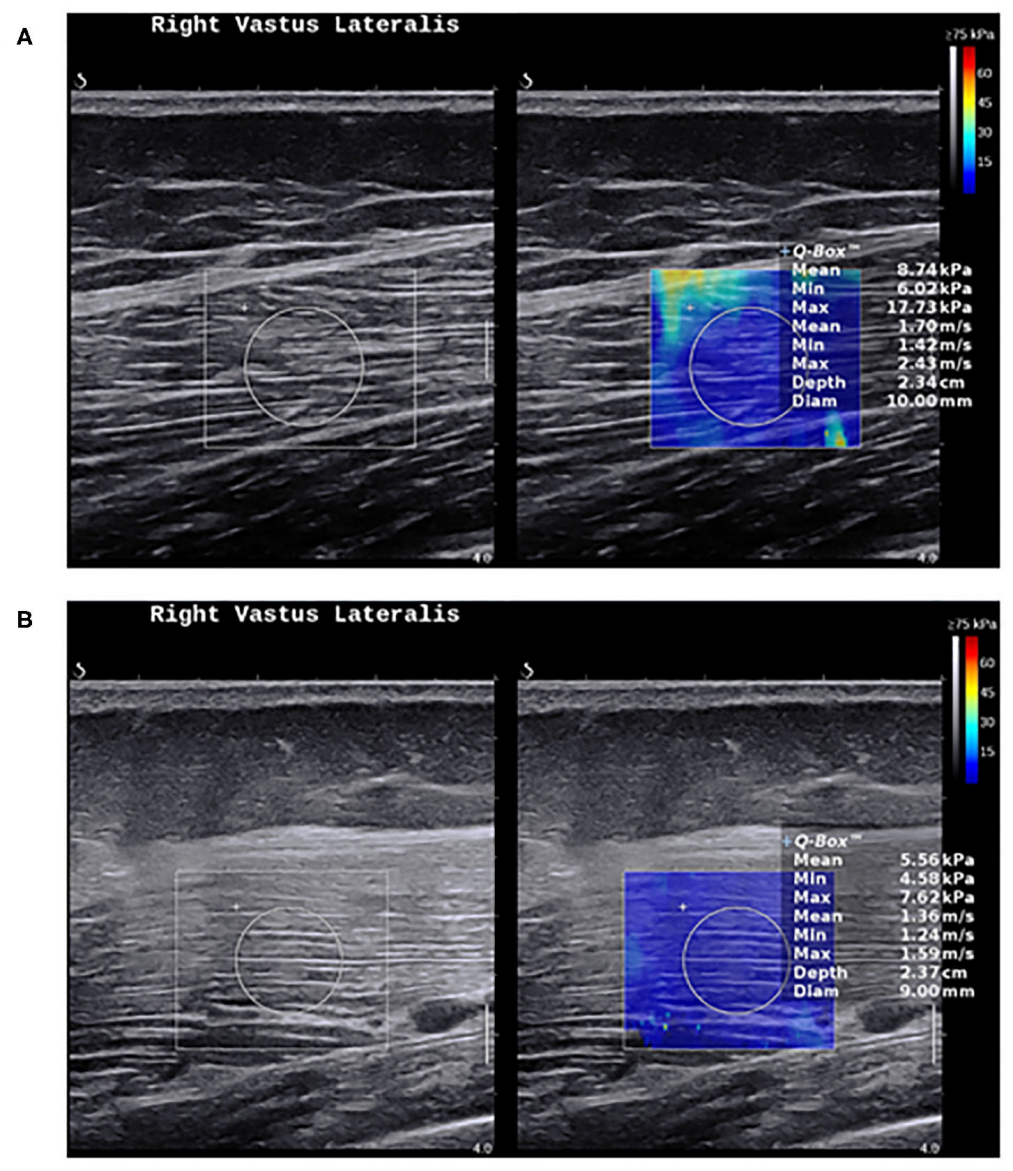
Shear wave elastography in healthy muscles and myositis. **(A)** Shows a normal muscle stiffness (8.7 kPa) in a 50-year-old healthy female person. **(B)** Shows a low muscle stiffness (5.5 kPa) in a 49-year-old male with active polymyositis.

Nevertheless, the current evidence suggests that SWE shows less muscle stiffness in myositis compared to healthy individuals (Figure 3), and can distinguish myositis from normal muscles (86). The loss of muscle stiffness in myositis patients was also observed using magnetic resonance elastography (87). SWE measurements also correlate with muscle strength and MRI grades of edema and atrophy (86, 88). All of these findings appear to only manifest when the muscles are under no passive or active loading.

IIM can be a very disabling condition. The potential to use promising non-invasive diagnostic and monitoring tools like qMRI and SWE could facilitate prompt diagnosis and treatment for patients. In the diagnosis of giant cell arteritis (GCA) (89), ultrasound can now reliably replace invasive temporal artery biopsies in GCA diagnosis. Similarly, the continuing development of qMRI and SWE of muscle could 1 day replace muscle biopsy in the diagnosis of IIM.

### Steroid Myopathy

Glucocorticoids are powerful anti-inflammatory agents and have a variety of uses in rheumatology, most commonly as bridging therapy before other longer term treatments are started. Polymyalgia rheumatic (PMR) and the related GCA are two examples where high doses of steroids are prescribed. As a result, many often develop a proximal myopathy, without typical inflammatory laboratory markers, such as muscle enzyme abnormalities or myositis-related antibodies. These patients are often disabled by muscle weakness from the disease process. It would, therefore, be reasonable to hypothesize that, despite not demonstrating a classical myositic picture with abnormal blood markers, muscles in PMR and GCA are likely to be abnormal.

There may be a fine line between the effects of inflammation from disease (PMR and GCA) and the catabolic effects of therapy (steroids) on muscle in patients. Amongst the many adverse effects of glucocorticoids, they trigger muscle atrophy, with a particular affinity for the atrophy of fast-twitch or type II muscle fibers (90, 91). This will often present as myopathy or muscle weakness, but due to the lack of a standardized definition of glucocorticoid-induced myopathy, reporting of myopathy due to therapy in PMR and GCA can prove inconsistent (92). Therefore, the management of steroid-induced myopathy can be challenging due to the difficulty in identifying myopathy before any clinical symptoms with the current means of investigation (93).

Can imaging help in characterizing the myopathy in this group of patients? Very little research in this area has been performed. Most studies have been focused on the diagnosis of PMR and GCA and responses to steroid therapy, based mainly on joint findings (94–97). Certainly, studies have demonstrated that quantitative ultrasound was able to show muscle changes associated to chronic use of steroids, but was unable to tell if the observed changes could be due to other causes including cachexia or sarcopenia (98, 99). A recent study showed that SWE detected a higher reduction in muscle stiffness over time in GCA patients on long term glucocorticoid who were also weaker (100). However, as patients with GCA (and PMR) tend to be older, and therefore more likely to be sarcopenic, these observed muscle changes have to be interpreted cautiously. If future research shows that SWE changes could potentially be evident before patients present with signs of weakness, then we may have an imaging tool that can direct appropriate management of steroid-induced myopathy, including preventative strategies to preserve muscle function.

The fact that type II muscle fibers tend to be affected by steroid therapy suggests that techniques like MRI diffusion tensor imaging that are sensitive to changes in muscle microstructure could be potentially useful in understanding the pathogenesis of steroid-induced myopathy and its diagnosis (80, 101). Due to the inflammatory nature of PMR and GCA, T2 MRI could be able to identify the edema within the muscle itself, which could be contributing to pain and fatigue. The muscle atrophy due to the catabolic effects of glucocorticoids could be quantitatively measured to monitor muscle change over time. The challenge will be interpreting the findings and to tease out if the observed imaging changes are due to therapy (glucocorticoids), or to the inflammatory disease process. Previously, when patients with RA were treated with long term steroids, it was possible to tell when they had weaker strength compared to patients who did not receive steroid therapy (102, 103). This would have provided a useful cohort to compare imaging findings of the muscle, and changes could be attributed to the steroid therapy independent of the disease process. However, due to the complexities of modern therapy and the ethical limitations, such direct comparison studies may not be feasible. Another iatrogenic cause of myopathy is in IIM treated with glucocorticoids, which presents another challenging dilemma in differentiating between muscle changes due to therapy and that due to the inflammatory muscle disease *per se*. This proposes an unmet need for means to identify the exact cause of the myopathy to optimize management—an area for further exploration of imaging as a potential tool for this purpose.

Nevertheless, qMRI can differentiate the muscle properties between the ages and has been shown to correlate with muscle outcome measures. Therefore, it shows potential promise as a tool to help understand the varying factors that can affect muscle in rheumatic diseases (104).

### Rheumatoid Arthritis

The predominant site of pathology in rheumatoid arthritis is in the joints. The articular joints are therefore the most commonly imaged structure in RA. However, there are many reasons for patients with RA to have weaker muscles, including impaired physical function and a greater tendency toward physical inactivity (105, 106). RA patients often present with lower muscle mass (107), which remains apparent in remission (105). A large proportion of RA patients report experiencing muscle problems or myopathy (108, 109). Histologically, RA is also associated with atrophy of type II muscle fibers, similar to the effects of glucocorticoids on muscle (110, 111). In addition, the pro-inflammatory state in inflammatory arthritis predisposes patients to a cachectic body composition—another reason for abnormal muscles in inflammatory arthritis (112, 113).

Despite the many causes of muscle involvement in inflammatory arthritis, there are relatively little muscle imaging data in RA. Reduced muscle strength is associated with disease activity in RA, and muscle function and physical activity are modifiable factors (2). Preliminary SWE of the muscles in RA shows some indication that muscles are less stiff compared to healthy individuals, but the results do not show statistical significance despite the fact that RA patients show reduced strength (114). The lack of differential findings from SWE studies suggests that muscle pathology in RA is less likely to be due to biomechanical properties of muscle. Quantitative MRI offers a different imaging perspective of muscle, and can provide further insight into the pathogenesis of muscle pathology in RA. Indeed, qMRI could be used to identify if rheumatic patients in remission still have muscle pathology, such as inflammation or fatty infiltration. This would identify whether effective treatment is improving muscle health, or if additional interventions, such as exercise, should be developed for a more holistic approach in patients with inflammatory arthritis.

Fatigue is a common symptom in many rheumatic conditions including inflammatory arthritis with significant impact on patients' lives (115). Although treatment including biological therapy can help improve symptoms of fatigue, they are not effective in all patients (116). Of note is that exercise has also been shown to reduce fatigue levels in RA (117); this suggests that modifying the muscles in inflammatory arthritis is a potential route to improving symptoms in patients. This is an area where the mechanism of action needs clarifying—an important cue for imaging, such as SWE and qMRI to help provide some insights.

## Future Perspectives and Conclusions

The capabilities of novel imaging in muscle continue to be stretched to better understand the significance of the observations. Quantitative ultrasonographic techniques, such as muscle echo-intensity may reveal useful imaging biomarkers beyond the mechanical properties of SWE (88). The use of both SWE and qMRI in assessing muscles are relatively recent imaging advances. Due to the heterogeneous nature of muscle involvement in rheumatic diseases, a multi-parametric imaging approach may offer a clearer picture of the varying disease processes (118). Combining both techniques could result in a more powerful imaging combination that provides complementary understanding of muscle changes (119). The application of artificial intelligence (AI) in imaging in rheumatology has enhanced efficacy and efficiency in image interpretation (120). Unsurprisingly, AI in rheumatology imaging is currently confined to assessing the common joint abnormalities like joint synovitis, tenosynovitis, bone erosions and cartilage loss. Deep learning involving qMRI and SWE may accelerate the knowledge and application of these imaging techniques.

This perspective highlights that the involvement of muscle is widespread in many rheumatic diseases, which can also affect other conditions including the connective tissue diseases like systemic lupus erythematosus, Sjogren's syndrome and systemic sclerosis (1). Imaging, in particular the more recent novel techniques like SWE and qMRI, shows potential to improve the understanding of how muscle is affected in rheumatic diseases. Imaging has an important role in assessing potential interventions on preserving muscle function. Imaging has improved our knowledge of joint abnormalities, but it is now timely for a call to action for a more anatomically-holistic approach toward the understanding of the pathogenesis of rheumatic diseases, with due attention to the muscle.

## Data Availability Statement

This research is funded by the NIHR infrastructure at Leeds. The views expressed are those of the authors and not necessarily those of the NHS, the NIHR or the Department of Health.

## Author Contributions

MF and AT wrote the first draft of the manuscript. JB, RW, and AA edited and revised the manuscript. MF, JB, and AA contributed the figures. All authors revised the manuscript critically and approved the final version of the manuscript. All authors contributed to the article and approved the submitted version.

## Conflict of Interest

The authors declare that the research was conducted in the absence of any commercial or financial relationships that could be construed as a potential conflict of interest.
